# Ultrasound-guided continuous femoral nerve block: a randomized trial on the influence of femoral nerve catheter orifice configuration (six-hole versus end-hole) on post-operative analgesia after total knee arthroplasty

**DOI:** 10.1186/s12871-018-0648-8

**Published:** 2018-12-19

**Authors:** Alessandra Novello-Siegenthaler, Mehdi Hamdani, Irène Iselin-Chaves, Roxane Fournier

**Affiliations:** 1 Department of Anesthesiology, University Hospitals of Geneva, Rue Gabrielle-Perret-Gentil 4, CH-1211 Geneva 14, Switzerland; 2 Clinique Générale-Beaulieu, Geneva, Switzerland

**Keywords:** Catheter orifice configuration, Continuous femoral block, Total knee arthroplasty, Local anesthetic consumption

## Abstract

**Background:**

Multiorifice catheters have been shown to provide superior analgesia and significantly reduce local anesthetic consumption compared with end-hole catheters in epidural studies. This prospective, blinded, randomized study tested the hypothesis that, in continuous femoral nerve block (CFNB) under ultrasound guidance, multiorifice catheter would reduce local anesthetic consumption at 24 h compared with end-hole catheter.

**Methods:**

Eighty adult patients (aged ≥18 years) scheduled to undergo primary total knee arthroplasty under a combination of CFNB, sciatic nerve block and general anesthesia were randomized to CFNB using either a 3-pair micro-hole (Contiplex, BRAUN®, 20G - 400 mm) or an end-hole (Silverstim VYGON®, 20G - 500 mm) catheter. Once the femoral catheter was sited, a bolus of 20 mL lidocaine 1% was injected. An electronic pump then delivered an automated 5 mL bolus of ropivacaine 0.2% hourly, with 10 mL self-administered patient controlled analgesia boluses.

**Results:**

There was no inter-group difference in either median number of ropivacaine boluses on demand during the first 24 h (4(2–7) vs. 4(2–8) in six-hole and end-hole groups, respectively; *P* = 0.832) or median ropivacaine consumption at 48 h (365(295–418) vs. 387(323–466); *P* = 0.452).

No significant differences were recorded between the groups at 24 h regarding median average verbal rate pain scale (2(0–3) vs. 2(0–4); *P* = 0.486) or morphine consumption (0(0–20) vs. 0(0–20); *P* = 0.749). Quadriceps muscle strength declined to 7% (0–20) and 10% (0–28) in the six-hole and end-hole groups, respectively, at 24 h after surgery (*P* = 0.733).

**Conclusions:**

In this superiority trial, catheter orifice configuration did not influence the effectiveness of CFNB in this setting: quality of analgesia was similar, with no reduction in either local anesthetic or morphine consumption, and equivalent postoperative quadriceps weakness.

**Trial registration:**

Retrospectively registered at (NCT03376178). Date: 21 November 2017.

## Background

Total knee arthroplasty (TKA) results in significant postoperative pain and continuous femoral nerve blockade (CFNB) is often provided for analgesia following such procedures [1–3]. One limiting factor of CFNB is motor weakness of the quadriceps muscle which may limit mobility [4] delay rehabilitation and may increase the risk of falling, particularly in elderly patients [5]. Various strategies have been tested to reduce the intensity of motor weakness while ensuring appropriate analgesia but none have yet succeeded [6, 7]. Bauer & colleagues evaluated the effect of varying the concentration and volume of local anesthetics (LA) on motor block intensity of quadriceps muscle with a continuous femoral block for bilateral TKA [7]. Their results suggested that the total dose of LA is the main factor that determines the effects on the femoral nerve [7]. Other studies have investigated whether different LA infusion regimens increase the efficacy of regional anesthetic techniques. For example, automated regular boluses (ARB) provided superior analgesia compared to continuous infusion in obstetric analgesia [8] and in some continuous peripheral blocks [9, 10]. In addition, ARB improved patient satisfaction and resulted in better differential blockade, with reduced LA consumption. A previous volunteer study [11] that compared two methods of LA delivery (basal infusion versus repeated boluses) in a femoral catheter failed to demonstrate any difference in quadriceps motor block; no mention was made about the number of holes used to deliver the LA.

Improved analgesia has been demonstrated in epidural studies with multiorifice catheters [12–14]. However, few studies have investigated the impact of catheter orifice configuration on the effectiveness of continuous peripheral nerve blockade. To our knowledge only two studies in the interscalene area have been published [15, 16]. In the first study, Frederickson [15] compared three threading distances in the interscalene groove with two types of catheters: one end-hole and two multiorifice. The results suggested higher average pain with the end-hole catheter but as its threading distance was the shortest it may be that an early displacement of the device could explain these findings, rather than the orifice configuration. In the subsequent study, three different catheters (end-hole, 3-hole and 6-hole) were inserted at the same depth; there were no differences between catheter groups in terms of analgesia [16].

It has to be pointed out that a low flow rate through a multiorifice catheter results mostly in proximal hole spread [17]. It is then mandatory to use an ARB and not a continuous infusion to deliver local anesthetic through all holes.

As the anatomical configuration appears to influence the infusion regimen of LA, the objective of this study was to compare the analgesic effect of an end-hole versus a six-hole (three pairs of micro-holes) femoral catheter placed under ultrasound guidance in the perioperative setting of a TKA. The primary study endpoint was to test the hypothesis that a multiorifice catheter would provide similar analgesia, but reduce total LA consumption at 24 h, compared with an end-hole catheter. Secondary endpoints were total LA consumption at 48 h, number of boluses at 24 h and 48 h, catheter visualization, quadriceps strength, need for rescue morphine, pain assessed on a numeric rating scale (NRS), and patient satisfaction.

## Methods

This study is reported according to the CONSORT (Consolidated Standards of Reporting Trials) guidelines [18].

### Trial design

The study protocol was approved by the local ethics committee (University of Geneva, Switzerland) with the number of Approval 12–269 and the ClinicalTrials.gov Identifier is NCT03376178.

Oral and written informed consent was obtained from all subjects before enrollment. The single-center, prospective, randomized, double-blind trial was designed with 1:1 allocation using computer-generated randomization.

### Participants

The study was conducted in the orthopedics department of the University Hospital of Geneva, Switzerland, over an 22-month period from May 2013 until April 2015. We prospectively enrolled adult patients (aged > 18 years), American Society of Anesthesiologists (ASA) physical status 1 to 3, scheduled to undergo elective primary TKA under general anesthesia combined with continuous femoral blockade and single sciatic nerve block under ultrasonographic guidance. Exclusion criteria were pregnancy, any contraindication to peripheral nerve blockade, pre-existing peripheral nerve neuropathy, allergy to LA (study medications), ASA score ≥ 4, neurologic or neuromuscular disease, psychiatric disease, renal failure, hepatic failure, chronic opioid therapy, NSAID contraindication, inability to use a patient controlled analgesia (PCA) device, genu valgum, infection at the injection site or withdrawal of consent.

### Randomization

Eighty patients were randomly assigned to CFNB in one of two groups. The first group (the six-hole group) were allocated to a tapered tip catheter with three pairs of lateral eyes, i.e. a six-hole catheter (Contiplex S Ultra, BRAUN R, 18G-100mm). The second group (the end-hole group) were allocated to an end-hole, open-tip silver coated echogenic catheter (Silverstim VYGONR, 18G-85mm).

The randomization sequence was computer generated (random.org) using a 1:1 allocation ratio, without any stratification or blocking. The random sequence was converted into sealed and opaque envelopes which were opened just before catheter placement. The practitioner who enrolled the patient (one of the authors) performed the insertion of the femoral catheter and the general anesthesia.

### Blinding

All investigators, patients, and other clinical staff were blinded to the treatment group. The investigator in charge of the patient was unaware of patient allocation. He did not perform the block or open the sealed and opaque envelope, but followed the patient in the ward. An opaque skin dressing was used in order to blind the type of catheter used. End-hole catheter Vygon is white and stiff whereas six-hole catheter contiplex is yellow and flexible.

### Preoperative procedures

Standard monitors were applied (noninvasive blood pressure, electrocardiogram, SpO_2_) and an intravenous access was established. Maximal voluntary isometric contraction (MVIC) of knee extension was assessed in all subjects at baseline with an electronic dynamometer (MicroFET2; Hoogan Industries, West Jordan, Utah) according to a previously published protocol [19]. The patient was seated with knees flexed at 90 degrees and the dynamometer placed perpendicular to the distal tibial crest. Patients were asked to extend the knee with a maximal force for 3 s while the assessor exerted an equivalent isometric force. Each measurement was taken three times sequentially, and the mean determined.

Midazolam and fentanyl were then titrated for comfort, while ensuring that patients remained responsive to verbal stimulation, with supplemental oxygen by face mask. Blocks were then performed as described below.

Continuous femoral nerve blockade was performed under supervision of anesthesiologists with substantial expertise in US-guided peripheral nerve blockades. We used an anterior in-plane approach with 13- to 6-MHz linear US probe (SonoSite S-nerve Fugifilm; Sonosite, Bothell, Washington) and a nerve stimulator in sentinel mode (1 mA, 2 Hz, 0.1 ms,). A sealed envelope was opened before the procedure; according to randomization, either an 18G 85 mm needle (Silverstim 85 mm-30 VYGON Ecouen, France) or 18G 100 mm needle (Contiplex S Ultra BBraun, Melsungen, Germany) connected to nerve stimulator (Stimuplex HNS 12; BBraun, Bethlehem, Pennsylvania) was inserted at the inguinal crease and orientated in a latero-to -medial in plane approach.

After sterile preparation (Betaseptic and isopropyl alcohol) and large inguinal crease draping, with the US probe in a sterile sleeve or cover (CG medical, Ternand, France), we proceeded to hydrolocalization with 5–10 mL 5% dextrose in order to open the space between the femoral nerve and the superior part of the ilio-psoas muscle. If a motor response of less than 0.5 mA appeared, the needle was repositioned until disappearance of the twitch. The catheter was then inserted under control and its position assessed between the femoral nerve and the superior aspect of the ilio-psoas muscle. Some aliquots of 5% dextrose were injected to confirm correct dispersal of the solution along the femoral nerve. The catheter was then secured with a stabilization device (Statlock, Bard Limited, Forest House, Sussex, UK) and a transparent Opsite (Smith-Nephew, France) dressing and then connected to the insertion port with an antimicrobial filter.

In both groups 20 mL of 1% lidocaine (Rapidocaine®, Sintetica, Mendrisio, Switzerland) were immediately injected through the catheter via 5 mL increments. The flow rate expected was > 400 mL/hour.

After 5 min, block assessments were carried out by a blinded member of the staff who was not involved in the block procedure. Progression of the sensory block from the antero-medial part of the leg to the anterior surface of the thigh was assessed using an icepack and compared with the contralateral leg every 5 min for 30 min (graded as 0 = normal sensation, 1 = blunted sensation, and 2 = absence of sensation). Motor blockade was evaluated considering knee extension using a 3-point scale: 0 = no motor block, 1 = partial motor blockade, and 2 = complete motor blockade. The block was considered successful if the sensory block in femoral distribution was complete within the first 30 min. If not, the patient was excluded from the study.

All patients received a single-shot ultrasound guided sciatic nerve block with an anterior approach using a 3–5 MHz curvilinear probe (SonoSite S-nerve, Fugifilm; Sonosite, Bothell, Washington) with nerve stimulator (Stimuplex HNS 12; BBraun, Bethlehem, Pennsylvania) in sentinel mode set to detect needle to nerve contact with a stimulus delivered at current of 1 mA, frequency of 2 Hz, and pulse duration of 0.1 millisecond. A Stimuplex D plus needle (120 or 150 mm, 22G) was orientated to the sciatic nerve antero-posteriorly with an in-plane approach to obtain a tibial twitch. If the motor response appeared between 0.5 and 1 mA, 20 mL of 0.5% levobupivacaine (Chirocaine®, Abott, Ringis Cedex, France) were injected through the needle. Time to sensory and motor blockade of sciatic nerve was tested by a blinded observer.

Patients then received standardized general anesthesia using propofol (1.5 to 2.5 mg/kg, sufentanil 0.3 mcg/kg and rocuronium 0.6 mg/kg). Anesthesia was maintained with sevoflurane approximately 1% in a 50% mixture of nitrous oxide and oxygen.

Before the surgical procedure, infusion pumps of 0.2% ropivacaine prepared by independent nurses and blinded to the investigative staff were connected to the femoral catheter. In both groups the infusion regimen was a combination of ARB of 5 mL every hour and PCA with an electronic pump (GemStar™, Hospira, Meudon La Forêt, France) consisting of self administered 10 mL boluses (on demand) with a lockout time of 60 min (bolus flow rate = 100–150 mL/hour). The pump delivered the first automated bolus immediately after catheter connection.

### Intraoperative management

The perioperative period was managed by a blinded anesthesiologist and titration of fentanyl was at his discretion.

### Postanesthesia care unit management

After surgery, patients were taken to the post-anesthesia care unit (PACU) where usual and systemic analgesics were provided. These included the perineural ropivacaine infusion initiated in the operating room, which continued until 48 h post-surgery, as well 7 days of oral acetaminophen (1 g every 6 h) and ibuprofen (400 mg every 8 h).

The verbal rating pain score was assessed (where 0 = no pain, 10 = worst imaginable pain), and this was considered time zero. All patients received instructions how to use the PCA and were monitored for signs of LA toxicity. If pain above the knee was present, the patient was encouraged to use the ropivacaine PCA. If no relief was observed after 30 min, 0.3 mg/kg oral morphine was administered. In case of sciatic blockade failure, the subject was excluded from the study. In addition, the aspect of the perineural catheter’s dressing was observed.

### Ward Management

All patients were discharged from PACU with regular administration of oral acetaminophen 1 g every 6 h, oral ibuprofen 400 mg every 8 h, and the perineural ropivacaine 0.2% infusion as required. If the pain score was ≥3 despite these analgesics, rescue medication of 0.3 mg/kg oral morphine was added.

The protocol study continued for 48 h post-surgery. The catheters were removed by nurses on the morning of the third postoperative day.

### Outcomes

Data collection was performed by an independent observer blinded to group allocation. The primary endpoint was ropivacaine consumption at 24 h.

Secondary end points included: the average and maximum pain scores 24 and 48 h after surgery (subjectively expressed by the patient) and measured on 10-point pain numeric rating scale (NRS), (where 0 = no pain, 10 = worst imaginable pain); number of ropivacaine boluses requested and delivered at 24 and 48 h; ropivacaine consumption at 48 h; morphine requirements; percentage of the preblock value of MVIC of quadriceps muscle, and the occurrence of complications or adverse effects. We observed patients’ ability to mobilize to a chair at 24 h and to walk between parallel bars at 48 h.

Secondary failure was defined as catheter dislocation visualized by ultrasound, or catheter dislodgement. At the end of the study, patients were asked to rate the quality of analgesia using a 10-point numeric rating scale (where 0 = unsatisfactory, 10 = very satisfied).

### Sample size

To calculate the required study sample size, we considered that the advantage offered by a multihole catheter would be clinically relevant if it resulted in a decrease in total local anesthetic consumption by about 20% compared with an end-hole catheter. Standard deviation of ropivacaine consumption through a femoral catheter was about 20% in a study by Ghandi [20], but we considered 25% to be closer to our everyday practice according to our previous findings with interscalenic catheters [21].

The minimum sample size required was 34 patients per group for a power of 90% with a 2-tailed significance level of 5% (β = 0.1 and α = 0.05). We therefore decided to enroll 80 patients, 40 in the six-hole group and 40 in the end-hole group, to allow for possible dropouts.

### Statistical methods

Data distributions were analyzed using the Kolmogorov- Smirnov test. Normally distributed data were compared between groups using the unpaired Student t test, and continuous variables with a non-Gaussian distribution were presented as median with ranges and compared between groups using Mann-Whitney U test. Group differences with nominal variables were analyzed using χ2 for proportions. Data are presented as mean ± SD, median with ranges, or percentage of patients. Statistical significance was defined as *P* < 0.05. The entire analysis was performed using the Statistical Package for the Social Sciences (SPSS for Windows, version 14.0; SPSS Inc., Chicago, Illinois).

## Results

### Participant flow

**Fig. 1:**
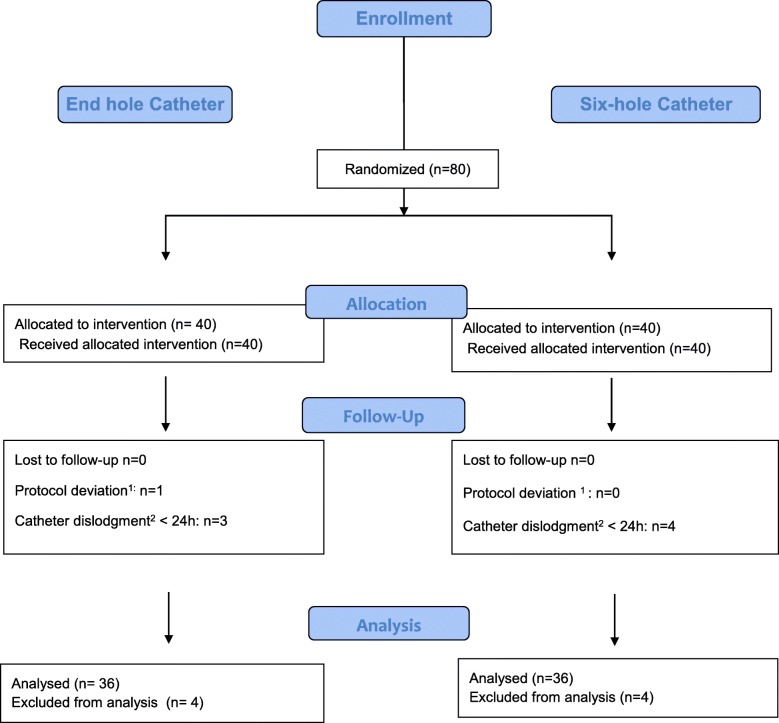
Patient flow through the study. One patient^1^ in the end-hole group had incorrect programming of the study pump. Three patients^2^ in the end-hole group and four patients in the six-hole group experienced catheter dislodgment on day of surgery

Eighty patients scheduled for elective primary TKA were enrolled in the study, 40 in the end-hole group and forty in the six-hole group. In the PACU or during the first night, three patients in the end-hole group and four in the six-hole group were excluded from the study because of catheter dislodgments. One patient in the end-hole group did not follow the bolus analgesia instructions correctly; thus, 72 patients completed the study, 36 in the end-hole group and 36 in the six-hole group (Fig. 1, CONSORT flow diagram).

### Recruitment

Enrollment started in May 2013 and ended in April 2015.

### Baseline data

Patient and surgical characteristics were similar in both groups (Table 1).

**Table 1:** Demographic Data of Both Groups

	End-hole *n* = 40	Six-hole *n* = 40	*P* value
Age (years)^a^	69 ± 7	70 ± 9	0.349
Gender (M/F)	18/22	16 /24	0.512
Weight (kg)^a^	85 ± 15	81 ± 19	0.309
Height (cm)^a^	168 ± 11	166 ± 11	0.539
ASA physical status (I/II/III)	0 /34 /6	1 /36 /3	0.442
BMI	31 ± 5	30 ± 5	0.982
Duration of surgery (min)^a^	140 ± 35	130 ± 24	0.128
Duration of anesthesia (min)	223 ± 59	197 ± 57	0.962

_a_Data presented as mean ± SD or percentage. *ASA* American Society of Anesthesiology

### Primary outcome

There was no difference in total local anesthetic consumption between the two groups either at 24 or 48 h postoperatively. Median number of boluses were also similar at 24 and 48 h in both groups (Table 2).

**Table 2:** Anesthetic and Analgesic Outcomes of Both Groups

	End-hole *n* = 36	Six-hole *n* = 36	*P* value
PRIMARY OUTCOME			
Ropivacaine volume at 24 h (mL)	178 (150–210)	173 (150–195)	0.478
SECONDARY OUTCOMES			
Ropivacaine volume at 48 h (mL)	387 (323–466)	365 (295–418)	0.452
Ropivacaine boluses at 24 h	4 (2–8)	4 (2–7)	0.832
Ropivacaine boluses at 48 h	5 (3–9)	7 (1–10)	0.768
Time of catheter placement (min)	20 (12–20)	15 (12–20)	0.751
Quadriceps strength at 24 h (%)	10 (0–28)	7 (0–20)	0.733
Quadriceps strength at 48 h (%)	31 (3–45)	16 (10–30)	0.394
Morphine consumption at 24 h (mg)	0 (0–20)	0 (0–20)	0.749
Morphine consumption at 48 h (mg)	20 (0–40)	20 (0–40)	0.785
No rescue morphine at 24 h	20	20	1
No rescue morphine at 48 h	10	13	0.614
Catheter visualization	9 (8–10)	7 (3–8)	0.001
Median of average NRS at 0 h (time zero)	0 (0–5)	0 (0–5)	0.797
Median of average NRS at 24 h	2 (0–4)	2 (0–3)	0.486
Median of maximum NRS at 24 h	4 (2–6)	4 (2–5)	0.219
Median of average NRS at 48 h	3 (1–4)	2 (0–3)	0.307
Median of maximum NRS at 48 h	4 (2–6)	4 (2–5)	0.232
Satisfaction score	9 (8–10)	9 (8–10)	0.808

Data are presented as n (%) or median with IQR. NRS: pain assessed on numeric rating scale, Satisfaction score: satisfaction on numeric rating scale, where 0 = not satisfied at all and 10 = very satisfied

### Secondary outcomes

Maximum and average pain scores, opiate requirements, patient satisfaction, and technical problems related to the catheter did not differ significantly between the two groups either at 24 h or 48 h after surgery (Table 2). Number of patients requiring morphine for breakthrough pain were similar in both groups at 24 and 48 h (Table 2).

Compared to preoperative values, quadriceps muscle strength declined to 10% (0–28) in the end-hole group versus 7% (0–20) in the six-hole group (*P* = 0.733) at 24 h; at 48 h these had increased to 31% (3–45) versus 16% (10–30) (*P* = 0.394) respectively.

At 24 h, 72% of the end-hole group versus 90% of the six-hole group patients were able to sit on a chair (*P* = 0.139). At 48 h, 53% versus 70% of patients in the end-hole and six-hole group, respectively, were able to walk between parallel bars (*P* = 0.184).

No signs or symptoms of systemic toxicity were observed. However, one patient in the six-hole group presented a quadriceps paresis 6 weeks after surgery at his first postoperative surgical control. An electroneuromyogram showed 50% axonal loss of the femoral nerve at the inguinal level consistent with the femoral nerve block.

Although no intergroup difference was found for any pre-specified endpoint, we also noted the ultrasonographic catheter visualization in both groups, based on the echogenic features of each type of catheter (scale 0 = no catheter visible, to 10 = catheter perfectly visible). The result was 9 (8–10) for the end-hole catheter versus 7 (3–8) for the six-hole catheter, which was a statistically significant difference (*P* = 0.001). However, failure rate of both catheters were similar with 4 cases in the six-hole group vs. 3 in end-hole group (*p* = 0.692).

## Discussion

This randomized trial, with blinding of investigators, outcome assessors and participants, did not find evidence to support the hypothesis that multiorifice catheter in the femoral area spares local anesthetic or morphine consumption at 24 and 48 h in the perioperative setting of TKA when compared with an end-hole catheter.

Multi-orifice catheters have previously demonstrated analgesic superiority in epidural studies of patients in labor [12–14, 21], with a reduction of unilateral block [12], or local anesthetic sparing [12–14]. In thoracic epidurals, such catheters resulted in more anesthetized spinal segments [22]. However, in order to detect a clinical difference in analgesia between an end-hole and multi-orifice catheter attention must be paid to flow rate. Homogenous LA spread through all holes requires at least a minimum flow rate of 80 mL/h that can only be delivered with intermittent bolus regimens. A background infusion results only in proximal hole spread [17]. Therefore, multi-orifice catheters should be coupled with an ARB to generate higher pressure, resulting in better analgesia and LA spread [10, 23].

This is to our knowledge the first trial that compared the potential benefits of multi-orifice catheters over end-hole catheters in the femoral area. Frederickson previously compared three threading distances (0.5, 2.5, 5 cm groups) in two different types of interscalene catheters (end-hole vs. triple-orifice) [15], although the end-hole catheter was in fact a standard triple orifice Contiplex catheter cut 1.5 cm proximal to the catheter tip. With an out-of-plane technique and antero-lateral approach, the end-hole catheter was threaded 0.5 cm past the needle tip whereas the triple-orifice catheters were threaded 2.5 cm and 5 cm, respectively, past the needle tip. There was no check of the correct catheter position with regard to the nerve roots. In the recovery room, pain was higher in the end-hole group than in the two triple orifice groups (34% vs. 6% vs. 9%, *P* = 0.0003), as well as time to first pain, ropivacaine bolus, and tramadol consumption at 24 h. These results suggest that triple-orifice catheters provide superior analgesia, but we may simply consider end-hole interscalene catheter early migration. This area is exposed to contraction of the scalene muscles, whose respiratory function may hasten catheter displacement precisely when it is positioned in the upper part of the interscalene groove. No statistically significant differences in analgesia were observed between the 2.5 cm and 5 cm groups. The author concluded that the known performance benefits of multiorifice epidural catheters [12–14, 21] also applied to peripheral nerve catheters.

In order to address the limitations of this first study, Frederickson subsequently explored the analgesic performances of three types of interscalene manufactured catheters (end-hole, triple-hole and six-hole) with standardized threading distance [16]. The primary endpoint was recovery room pain after a bolus injection of 15 mL 0.375% ropivacaine through the three catheters. Secondary endpoints were time to first pain and tramadol consumption. None of the three endpoints differed between groups. In this study, it should be noted that the pump was set to deliver ropivacaine 0.2% 2 mL/h through the interscalene catheter, an administration regimen which does not guarantee a homogenous distribution of LA through all holes, as noted earlier. This may have affected the results. Moreover, the catheter was advanced 5 to 7 cm beyond the needle tip and then definitively positioned *blindly* 3 cm past the original tip position. In our study, we used intermittent bolus regimens in both groups. We also determined precisely the definitive location of our catheters under ultrasound guidance using an in plane short axis approach. The possibility of viewing the entire needle shaft and the nerve optimizes needle tip and catheter positioning. In our opinion this accuracy was important to gain precision and maximize analgesic benefits in both treatment groups. We chose to slip all our catheters under the femoral nerve and over the ilio-psoas muscle after crossing the fascia iliaca in order to minimize possible migration and obtain a homogenous spread of LA around the femoral nerve. However, despite all these precautions, we had a similar femoral catheter failure rate in both groups (5 to 10%). We did not observe any difference between groups in terms of local anesthetic consumption at 24 h and 48 h despite an appropriate methodology, with a standardized distribution of LA through all holes, and a similar, precise location of the tip of all study catheters. Our results may be explained by the location of our catheters: trapped between nerve and muscle, local anesthetic is pushed by pressure around the nerve irrespective of the number of holes. We may also add that in our study, continuous femoral blocks were only part of a multimodal strategy including sciatic nerve block and paracetamol plus NSAIDS on a daily base. It is then quite difficult to find a significant difference between groups. Systemic analgesia has probably help in masking differences between local anesthetic consumption.

Femoral nerve block is commonly used for analgesia in patients undergoing total knee replacement [1]. However, its main disadvantage is quadriceps weakness resulting in impaired mobility and fall risk [5]. In our study, we did not observe any significant differences between the end-hole and six-hole catheter in terms of quadriceps muscle strength. This finding appears logical given that total LA consumption at 24 and 48 h did not differ between groups. In this precise setting (in plane, short axis and femoral location), a six-hole catheter did not obviously spare motor function. However, there were no block-related patient falls. There was a trend to earlier rehabilitation in the six-hole group, which did not reach statistical significance, but our study may have been underpowered to detect that outcome.

Limitations.

Our study has limitations. We decided to exclude patients with failed blocks, both femoral and sciatic blocks, and perform per protocol analyses rather than intention to treat analyses. As the number of failures were low, we may hope that the potential attrition bias has not impacted the results. The anesthesiologist providing the perioperative care, ie continuous femoral block, sciatic nerve block and general anesthesia was unblinded, and this could have affected the results. However, nurses (recovery room and ward) and the investigator were unaware of patient allocation.

It should be noted that all our blocks were performed by trainees supervised by fellows, which is standard practice in our University Hospital. Our results are then marked by good external validity.

## Conclusions

In summary, our superiority study demonstrated that a six-hole femoral catheter did not provide any advantage in terms of local anesthetic consumption, or quadriceps motor function sparing, when compared to an end-hole catheter. This lack of advantage confirms previous findings in the interscalene area [16], but confirmatory randomized studies in other locations such as the sciatic nerve are still needed.
